# Nursing protocol in chronic kidney disease prevention in older adults in primary care

**DOI:** 10.1590/0034-7167-2022-0052

**Published:** 2022-11-28

**Authors:** Fernanda Ferreira Krepker, Cristina Arreguy-Sena, Luciene Muniz Braga, Paula Krempser, Jéssica de Castro Santos, Herica Silva Dutra

**Affiliations:** I Universidade Federal de Juiz de Fora. Juiz de Fora, Minas Gerais, Brazil; II Universidade Federal de Viçosa. Viçosa, Minas Gerais, Brazil

**Keywords:** Protocols, Nursing, Primary Health Care, Nursing Records, Renal Insufficiency Chronic, Protocolos, Enfermería, Atención Primaria de Salud, Registros de Enfermería, Insuficiencia Renal Crónica, Protocolos, Enfermagem, Atenção Primária à, Saúde, Registros de Enfermagem, Insuficiência Renal Crônica

## Abstract

**Objectives:**

to develop a protocol for Nursing Process operationalization in approaching older adults with vulnerability to chronic kidney disease in Primary Health Care, based on Neuman’s stressors.

**Methods:**

a methodological study, carried out in two stages: 1) synthesis of evidence using an inductive strategy (mixed method study) and 2) protocol development to support the nursing process operationalization with older adults enrolled in a Basic Health Unit, using a deductive strategy (Neuman’s stressor concepts, NANDA, NIC, and NOC taxonomies, Risner’s line of reasoning, and cross-mapping), described according to A Step-by-Step Guide to Developing Protocols.

**Results:**

102 older adults participated, and 17 diagnoses, 34 interventions and 26 nursing outcomes were identified.

**Conclusions:**

the protocol developed is a technology that makes it possible to operationalize the Nursing Process, based on Neuman’s stressors and on taxonomy, conceptual and care frameworks, guiding care and nursing records.

## RESUMO

**Objetivos:**

desenvolver um protocolo para a operacionalização do Processo de Enfermagem na abordagem de pessoas idosas com vulnerabilidade para doença renal crônica na Atenção Primária à Saúde, alicerçado nos estressores de Neuman.

**Métodos:**

estudo metodológico, realizado em duas etapas: síntese das evidências por estratégia indutiva (estudo de método misto) e desenvolvimento do protocolo para subsidiar a operacionalização do Processo de Enfermagem com pessoas idosas adstritas a uma Unidade Básica de Saúde, utilizando estratégia dedutiva (conceitos de estressores de Neuman, taxonomias NANDA, NIC e NOC, linha de raciocínio de Risner e mapeamento cruzado), descrito segundo *A Step-by-Step Guide to Developing Protocols.*

**Resultados:**

participaram 102 idosos. Foram captados 17 diagnósticos, 34 intervenções e 26 resultados de enfermagem.

**Conclusões:**

o protocolo elaborado constitui uma tecnologia que possibilita operacionalizar o processo de enfermagem alicerçado nos estressores de Neuman e em referenciais taxonômicos, conceituais e assistenciais, norteando o cuidado e os registros de enfermagem.

## RESUMEN

**Objetivos:**

desarrollar un protocolo para la operacionalización del Proceso de Enfermería en el abordaje de ancianos con vulnerabilidad a la enfermedad renal crónica en la Atención Primaria de Salud, a partir de los estresores de Neuman.

**Métodos:**

estudio metodológico, realizado en dos etapas: 1) síntesis de evidencias utilizando una estrategia inductiva (estudio de método mixto) y 2) desarrollo del protocolo para apoyar la operacionalización del proceso de enfermería con personas mayores inscritas en una Unidad Básica de Salud, utilizando una estrategia deductiva (conceptos de factores estresantes de Neuman, taxonomías NANDA, NIC y NOC, línea de razonamiento de Risner y mapeo cruzado), descritos de acuerdo con *A Step-by-Step Guide to Developing Protocols*.

**Resultados:**

Participaron 102 ancianos. Se capturaron 17 diagnósticos, 34 intervenciones y 26 resultados de enfermería.

**Conclusiones:**

el protocolo desarrollado es una tecnología que posibilita la operacionalización del Proceso de Enfermería, a partir de los estresores de Neuman y de referencias taxonómicas, conceptuales y asistenciales, de los cuidados orientadores y de los registros de enfermería.

## INTRODUCTION

The Brazilian Unified Health System (SUS - *Sistema Único de Saúde*) is structured at polyarchic levels organized by the Health Care Networks (RAS - *Rede de Atenção à Saúde*), operationalized at the levels of primary, secondary and tertiary health care, which is characterized by the formation of horizontal relationships between services, the search for a multiprofessional, continuous and comprehensive approach, the inclusion of health and economic perspectives and structured support and logistics systems according to technological density^(1)^.

The RAS structuring at the Primary Health Care (PHC) level for older adults is planned to run through the care network connection, in its levels of care focused on disease prevention and health promotion, aiming to meet their specificities of prevalence of chronic comorbidities, expressed by the triple burden of diseases (infectious, deficiency and external causes diseases), due to the demographic transition characteristic of the Brazilian aging process^(2)^.

However, elder care reflects a fragmented, reactive, episodic health system focused on coping with acute conditions and exacerbations of chronic conditions^(3-4)^. In this context, the vulnerability of older adults to chronic kidney diseases is inscribed, guided by the Brazilian National Policy for the Care of Patients with Kidney Disease (*Política Nacional da Atenção ao Portador de Doença Renal*), which establishes care strategies structured in an equitable, qualified care centered on prevention, promotion, treatment and rehabilitation actions^(5)^.

Among the therapeutic actions that can be developed in a Basic Health Unit (BHU), are the early detection of chronic diseases, comorbidity management, such as systemic hypertension (SH), diabetes mellitus (DM), autoimmune diseases and renal alterations operationalized by active search and home visits^(6)^. Chronic kidney disease (CKD) occurs among older adults with comorbidities and polypharmacy, intensifying the worsening or loss of kidney function, to the point of making it difficult for this segment to adapt to instabilities in fluid and electrolyte balance^(7)^. This fact justifies the structuring of nursing care, to the extent that CKD is a public health concern associated with high expenses and professional performance on exposure, installation and development.

The insertion of nurses and their team in PHC can be structured, in this context, based on the Nursing Process (NP), a scientific methodology used to guide and qualify nursing care, systematizing the care provided to users, through interrelated, interdependent and recurrent stages, namely: data collection; nursing diagnosis; nursing planning; implementation; and nursing assessment^(8)^.

In the PHC scenario, the coexistence of professionals with undergraduate and graduate degrees (*latu sensu* and *stricto sensu*) and the Family Health Strategy (FHS) favors that this scenario is conducive to the development of Advanced Nursing Practice, for which the construction of a nursing protocol can help and direct Brazilian nurses’ work in this context^(9)^.

To standardize language and nursing records, we chose to use the taxonomies of nursing diagnosis, interventions and outcomes (NANDA-I - taxonomy of diagnoses, Nursing Intervention Classification (NIC) of interventions and Nursing Outcome Classification (NOC) of nursing outcomes), because they allow consensus building and information sharing between professionals and services at an international and national level^(10-12)^.

Stressors are being conceived as situations/problems of intrapersonal, interpersonal and extrapersonal origin, generators of stimuli and producers of tensions and instabilities in the energy system (lines: flexible defense, normal defense and resistance) of older adults, which impacts on their energetic structure, whose identification of its origin, nature, intensity and reactions can guide nursing care and the search for energy balance (health)^(13)^.

In order to contribute to overcoming the gap in the structuring of nursing care using technologies (theoretical, taxonomic models and specialized technical knowledge) adaptable to PHC nurses’ clinical practice, this investigation has as its object the development of a protocol to operationalize the NP in the approach of older adults in PHC who are vulnerable to CKD based on Neuman’s stressors.

The following question arose: is it possible to develop a protocol to subsidize NP in PHC with older adults vulnerable to CKD based on theoretical-philosophical, taxonomic and legal frameworks?

The present investigation is justified on the basis of the following arguments: compliance with the Federal Nursing Council recommendations regarding the NP in the scenarios in which nurses work; need to base the PHC nursing team practice on philosophical, taxonomic and scientific frameworks that allow the exchange of experience between nurses who work at an international and national level; structuring of a protocol to guide students’ and nurses’ performance, when caring for older adults vulnerable to CKD, making it compatible with its computerization and adding problem-solving to the care in PHC.

## OBJECTIVES

To develop a protocol for NP operationalization in approaching older adults with vulnerability to CKD in PHC, based on Neuman’s stressors.

## METHODS

### Ethical aspects

This study met the ethical and bioethical requirements for conducting research with human beings, according to Resolution 466/2012 of the Brazilian National Health Council (*Conselho Nacional de Saúde*). After consideration by the Research Ethics Committee of the *Universidade Federal de Juiz de Fora*, the project was approved in 2019. Participation in the study was preceded by reading, agreement and signing of the Informed Consent Form (ICF) by all study participants.

### Study design, period, and location

This is a methodological study, built according to the MethodologIcal STudy reportIng Checklist (MISTIC) tool. The study scenario comprised the coverage area of a BHU, with an estimated population of 21 thousand inhabitants, in a city in the state of Minas Gerais with approximately 560 thousand inhabitants. Data were obtained between June 2019 and March 2020, with the participation of older adults in their respective homes.

### Sample; inclusion and exclusion criteria

The sample was gathered by convenience. We included older adults ≥ 65 years old and living on streets attached to a BHU. We excluded older adults who had speech or hearing impairments, who were absent during the data collection period or who postponed participation for more than five times, who did not participate in more than 50% of the investigation and did not agree to record the interview or take the tests.

### Study protocol

Study operationalized in two stages: 1) synthesis of evidence by inductive strategy (convergent parallel mixed method study)^(14)^; and 2) protocol development to subsidize NP operationalization with older adults enrolled in a BHU, using deductive strategy (concepts of Neuman’s stressors, diagnosis taxonomies, nursing interventions and outcomes, Risner’s line of reasoning and cross-mapping)^(10-13)^, described according to A Step-by-Step Guide to Developing Protocol^(15)^.

In the first step, the results of a convergent parallel mixed method study^(14)^ composed the evidence. An inductive strategy was used to synthesize the main problem situations that require therapeutic intervention and that serve as a foundation for nursing care. These were results from a qualitative step based on procedural^(16)^ (n=50) and structural^(17)^ (n=102) approaches of the Theory of Social Representations (TSR)^(18-19)^, using a guiding question (tell me your experience, what you heard or know about the possibility of an older adult having CKD) and inducing term “kidney disease-problem in older adults”.

In the quantitative stage (n=102), participants were characterized according to sex, age, education, self-reported diseases and medications, smoking, physical activity, blood pressure measurements, capillary blood glucose, Body Mass Index (BMI) and Clinical-Functional Vulnerability Index (IVCF-20)^(20)^, assessing associations with the outcomes of diseases and use of medications. The triangulation of this information supported the identification of problem situations that could be addressed at the PHC level of older adults vulnerable to CKD.

In the second stage, the construction of a protocol was performed, described according to A Step-by-Step Guide to Developing Protocols. Nine of the 12 steps of the protocol were used. The non-use of steps 10 (implement the protocol in the service), 11 (monitor variations) and 12 (review the protocol) occurred due to the emergence of the COVID-19 pandemic and the periods of recommendation of social isolation and interval blocking by which passed the investigation scene

Step 1: Theme selection and prioritization: in the present investigation, the eligible theme was nursing care, with people aged ≥65 years and vulnerable to CKD.

Step 2: Team assembly: from the activities developed with older adults in the research group Technology, Culture, Communication in Health and Nursing (TECCSE-UFJF - *Tecnologia, Cultura, Comunicação em Saúde e em Enfermagem*), studies were developed (2010 to 2021) on the aging process, considering that they were assigned to a BHU and targets of research, teaching and extension activities. This initiative motivated the deepening of the vulnerability of this population segment to chronic diseases and, in particular, to CKD (focus of the present investigation).

Step 3: User inclusion: studies on the concept of being older adults, having urinary and fecal incontinence, having comorbidities, being vulnerable to falls at home and around the home, assessing mental status and the occurrence of depression showed that some of these situations could be prevented, treated early or therapeutically addressed at the PHC level. This fact motivated researchers and professionals to approach people aged ≥65 years, creating a register that guided care actions carried out through activities related to undergraduate teaching, research projects linked to scientific initiation and dissertations of master’s degree and extension activities. These activities allowed the research group to be closer to users, creating a bond that ensured the intermediation of BHU with users, through at least three semiannual visits to each one of them.

Step 4: Protocol objective establishment: the protocol aimed at the elaboration of a technologically compatible tool for the identification of problem situations of older adults vulnerable to CKD, based on Neuman’s stressors, taxonomic, conceptual and care frameworks and national and international scientific evidence. This objective is justified by the coexisting morbidities identified among older adults (SH, DM, cardiac and autoimmune diseases and self-medication situations) and by the possibility of minimizing exacerbation events, emergence and worsening of these chronic diseases.

Step 5: Commitment and conscience creation: the research group approach with the BHU multidisciplinary team and the continuity of research, teaching and extension activities developed in the same scenario characterize a bond and the joining of efforts of all, in the sense of identifying a protocol that maximizes professional activities and provides solutions to the needs of BHU users who are older adults, their families and caregivers.

Step 6: Information gathering: the information used came from the following sources: 1) experience in the service; 2) empirical investigation from the synthesis of evidence using an inductive strategy (mixed method study); 3) taxonomy frameworks (diagnoses, interventions and nursing outcomes), as they allow consensus building and the sharing of information between professionals and services, nationally and internationally^(10-12)^, theoretical-philosophical frameworks (Neuman System Theory’s stressors)^(13)^ and thematic frameworks (guidelines from national and international evidence supported by the Brazilian Society of Nephrology on CKD prevention and management)^(6)^.

Step 7: Initial assessment: the meeting of a team of nurses with different activities and functions (professors, master’s, residency and undergraduate students) who had, in common, working with older adults, motivated the deepening of the theme of vulnerability of this population segment to CKD. The meeting of experts made it possible to identify the absence of a structuring protocol for therapeutic nursing actions in the context of the FHS of a BHU and that could be operationalized by the home visit, consultation carried out at the BHU or online consultation motivated by the COVID-19 pandemic^(21)^. Based on the mixed method study results, evidence was identified that could be addressed by nursing care, motivating identifying the focus of interventions evidenced in the investigated group and that can be addressed be systemic care. Considering the above, contents, scientific evidence, possible nursing problems, therapeutic actions and evaluative parameters were listed, using NANDA-I, NIC and NOC (NNN) taxonomies to express and standardize these elements, basing them on the protocol.

Step 8: Protocol production: the protocol, as a technological tool, was entitled “*Protocolo de enfermagem para o cuidado de pessoas idosas vulneráveis a DRC*- (PECPIV-DRC)”, being structured according to the legislation of the Federal Nursing Council on NP and Neuman’s concept of stressors^(13)^ in the context of vulnerability of older adults to CKD. The production of this protocol incorporated procedures such as: script for data collection (nursing history structured from stressors and human variables); a list of possible nursing diagnoses described according to the NANDA-I taxonomy; nursing outcomes described according to NOC taxonomy; and list of therapeutic actions and interventions according to NIC taxonomy.

Step 9: Pilot test application in the academy: the meeting of experts in care management, NP, use of a standardized nursing language system according to NNN taxonomies and elder care in the context of PHC allowed the consensus of components that underpinned the PECPIV-DRC protocol construction for the context of a BHU, whose parameters used as cut-off points: ≥90% consensus of fully in agreement and 80% for partially in agreement^(22)^.

### Analysis of results, and statistics

Data analysis of the qualitative stage used content analysis, categorical thematic (procedural approach) and elaboration of the four-box chart, treated by prototypical analysis and co-occurrence test, respectively (structural approach)^(17,23)^. Descriptive (frequency and percentage) and inferential (chi-square test) statistics were used to analyze the data from the quantitative stage. A p-value < 0.05 was adopted.

## RESULTS

A total of 102 older adults participated, characterized as follows: women (68.2%); age ≥80 years (35.2%); low education (<8 years - 70.5%); with one to three self-reported diseases and medications in use (57.9% and 43.3% respectively); self-reporting to be hypertensive (72.7%), with diabetes (21.6%) and dyslipidemia (25%); with changes in blood glucose levels by measuring capillary blood glucose (81.8%) and systolic (35.2%) and diastolic (15.9%) blood pressure; smokers (13.6%) and non-practitioners of physical activity (72.7%), with BMI ≥ 27 kg^2^ (36.4%). The IVCF-20 of 88 participants was also assessed, with a score from 0 to 6: risk of low clinical-functional vulnerability, score from 7 to 14: increased risk of vulnerability (35.2%) and score from 15 to 40: high risk of vulnerability (44.3%). A statistically significant association was identified between medication use and having children (p<0.001), education (p<0.001), age (p=0.044), hypertension (p<0.001) and physical activity (p<0.001); and self-reported disease and having a child (p<0.001), education (p<0.001), age (p=0.038) and physical activity (p=0.001).

**Figure 1:**
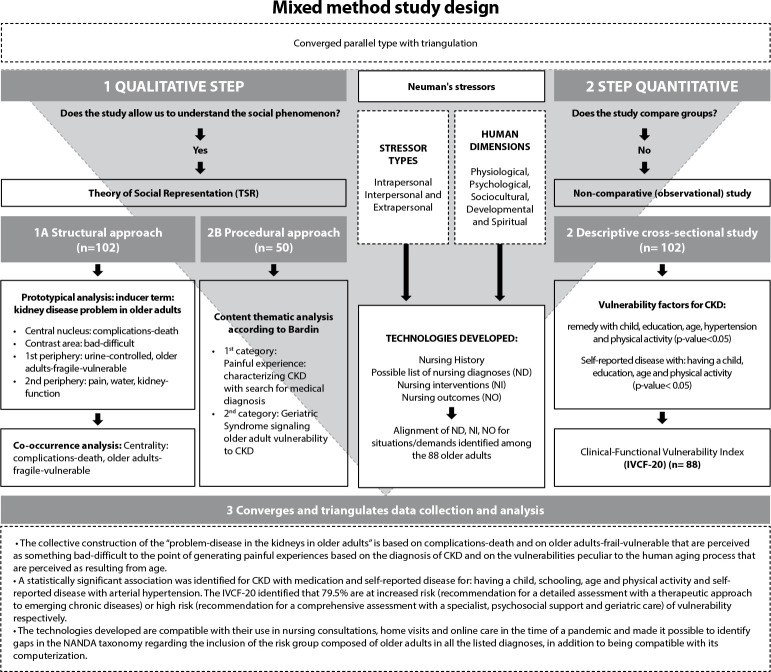
Triangulation process synthesis and mixed method study results

Figure 1 shows the mixed method study allowed triangulating the information.

*IVCF-20 was carried out with 88 participants, due to data collection interruption due to the COVID-19 pandemic.*

In the second stage, the PECPIV-DRC protocol construction was carried out, operationalized through a script for carrying out nursing history (NH), data collection, lists of possible diagnoses, outcomes and nursing interventions.

To support the data collection script elaboration, a semi-structured interview was planned with older adults vulnerable to CKD and/or their families/caregivers, aiming to obtain information about their health-disease process conditions. Such contents emerged from the sectional study outcomes in a layout with three vertical columns and five horizontal lines, allowing to accommodate Neuman’s stressors (intrapersonal, interpersonal and transpersonal) and the variables that integrate the concentric energy lines of the basic structure of human beings (physiological, sociocultural, psychological, developmental and spiritual). In this proposal, it is intended for data collection, made possible through nursing consultation (NC), home visit (HV) or virtual contact.

The possible nursing diagnoses were obtained by applying the technique of analysis (deliberation stage) and deduction (judgment and decision stages), proposed by Risner, making it possible to deduce from the content arising from the script for the NH the problem-situations of relevance to the professional competence of nurses and that portray the basic needs of older adults with vulnerability to CKD, arising from the triangulation of the mixed method study results.

The diagnosis cluster layout was presented in two columns: on the left, numbered incrementally, and on the right, containing its title and components (related factors, risk factors, associated conditions and risk populations). Seventeen possible nursing diagnoses were included, compatible with the problems identified in NH uptake, of which five focused on health promotion, five focused on risk, and seven focused on the ongoing problem.

For the selection of therapeutic activities compatible with the demands identified in the NH and named in the possible diagnoses selected, 35 interventions were listed with their respective therapeutic activities, according to the NIC taxonomy. It is worth mentioning that, among 79.5% of participants, there was the influence of clinical-functional vulnerability level, which made it possible to categorize them as having interpersonal stressors that require therapeutic support for activities of daily living or instruments of daily living. To explain the indicators and measurement scales to be used in nursing intervention assessment, according to the NOC taxonomy, a protocol was built with components compatible with the situations/problems listed. Twenty-four nursing outcomes were listed with their respective scales and measurement parameters.

It is worth mentioning that the intervention layout was structured in sequential lines, containing therapeutic interventions and respective therapeutic actions. The nursing outcome layout includes the intervention title, the scales used and the parameters presented in three independent columns.

Nursing diagnoses, interventions and outcomes were obtained using the cross-mapping technique, using NANDA-I, NIC and NOC taxonomies to express and name such situations. In order to operationalize the approximation between diagnoses, outcomes and interventions, an instrument was built that visually aligns the parameters and scales of the indicators with the therapeutic interventions and identified problem situations.

In structuring the layout of this instrument, it was foreseen its use in the same form, during eight subsequent consultations, portrayed in the eight squares that precede the list of nursing outcomes and interventions, whose completion allows the visualization and monitoring of health status and the therapeutic procedures. It is presented in three axes: 1) identification data; 2) alignment between the diagnosis titles, the outcomes to be parameterized and the recommended/compatible therapeutic interventions, with additional space provided for the inclusion of peculiar situations that emerge; and 3) place for registering the date, signature and professional stamp of each of the consultations carried out^(24)^.

It is worth mentioning that there is space available for new additions, modifications, suspension or cancellation of registered contents, adding dynamism to the process, favoring the monitoring of older adults’ health status dynamics and for identifying the professional responsible for each meeting (signature, record and date), with the possibility of its electronic use being foreseen.

**Figure 2:**
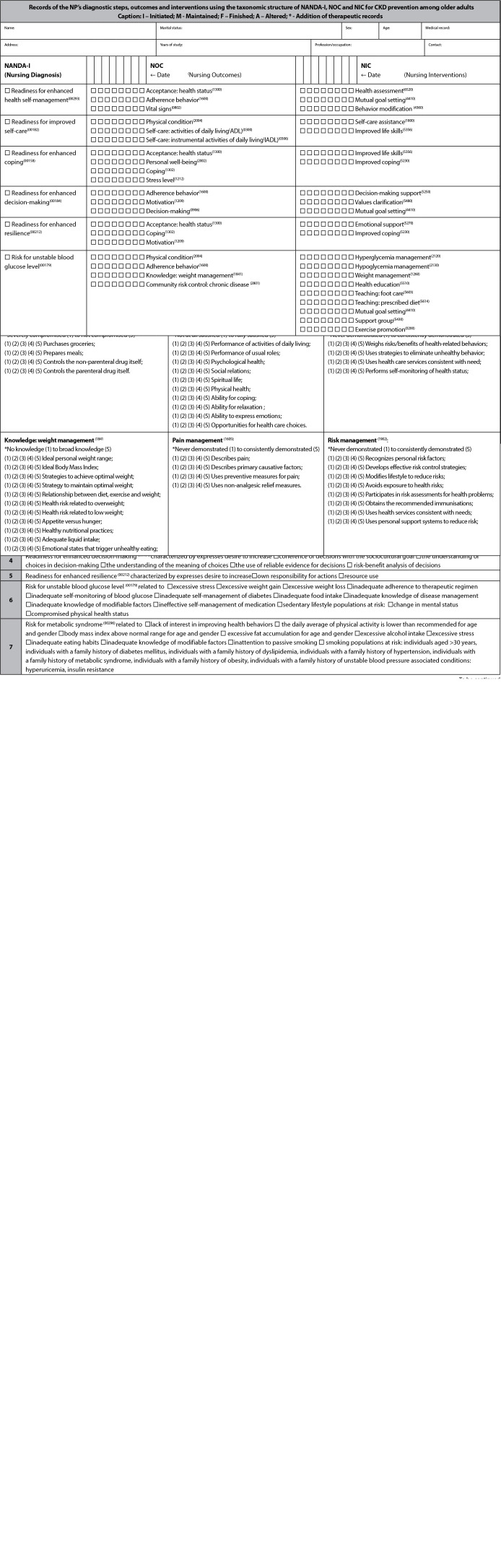
Example of the steps included in the PECPIV-DRC protocol

Figure 2 shows the example of the steps inserted in the complete PECPIV-DRC protocol.

*DIM - dimensions; PH - physiological; PS - psychological; SC - sociocultural; DV - developmental; SP - spiritual.*

## DISCUSSION

Personal and social characteristics were related to the occurrence of CKD in an epidemiological survey, which identified the prevalence of self-reported CKD in Brazil and characterized the associated factors. It corroborated the findings that support the protocol presented, by concluding that the prevalence of the disease was higher in people with more advanced age, low education, smokers, hypertensive, with hypercholesterolemia and regular or poor health status assessment, subsidizing actions for prevention and structuring of public health policies^(25)^. The prevalence of women in the study is supported by the feminization of aging, justifying the differences in the causes of morbidity and mortality, coverage of interventions, lifestyle habits, exposure to risk factors and determinants of the aging process among men and women^(26)^.

There is evidence that the decrease in glomerular filtration and kidney damage intensify with age and are secondary to chronic diseases, with greater access to information among younger people and better compliance with care among older people, which makes them susceptible to CKD. Low education impacts on lifestyle and preexisting diseases, affecting the level of information about the disease and treatment^(27)^.

In this sense, the approach to health promotion and disease prevention takes place in PHC, considered the gateway to the SUS, a priority space for the nursing team to work. At this level of care, the structuring of a protocol to subsidize nursing care for older adults with chronic diseases, including CKD, coincides with the lines of priority care determined by the Ministry of Health^(2-3,5)^.

There are recommendations for managing risk factors and comorbidities in PHC, such as SH, DM, use and nephrotoxic drug management, family history, anemia, bone and cardiovascular changes^(28)^. Low compliance with (inter)national KDIGO Guidelines for monitoring and reducing progression to CKD in PHC led to a cohort of 6,931 people who had a glomerular filtration rate <60 ml/min for 1.73 m^2^, recruited in four countries (France, USA, Germany and Brazil), assessing compliance with monitoring and deferring the progression of CKD. Differences were detected in albuminuria (Brazil (36%) and Germany and USA (43%) and in blood pressure levels between non-diabetic people and those without proteinuria (Brazil (76%) and France (49%), with parameters of lower normality in Brazil (52%), when compared to other countries (<40%). This occurred in a younger Brazilian age group (Brazil 65 years and Germany 72 years), when compared to other countries^(6)^.

The work activity of nurses with older adults at risk for developing CKD attached to a BHU requires that their professional performance be based on the Systematization of Nursing Care and the NP, which makes the outcomes of this investigation serve as a foundation for the identification of basic human needs and human responses, which can be modulated by nursing care. There is a recommendation from the Federal Nursing Council that the structuring of nursing care be based on theoretical framework^(8)^ and standardized taxonomies^(10-12)^.

The choice of Neuman’s theoretical-philosophical framework is justified because it addresses basic human needs, focusing on aspects of prevention of complications and health promotion, portraying a proposed approach to nursing that can be used by the health team. The detailed assessment of the impact of stressors on older adults’ energy system, when aligned with the skills provided in the training of nurses, guides therapeutic nursing interventions that can occur at the level of primary prevention, whose action may be triggered at any time when vulnerability or risk factors for CKD are identified to maintain balance in the system. At the secondary prevention level, the early discovery of changes in glomerular filtration may favor the referral to secondary health care, aiming at disease diagnosis and early treatment. At the tertiary prevention level, professionals working in PHC may engage in the prevention of complications and self-care, to avoid aggravation^(13)^, following the referral and counter-referral flow, in order to avoid losing the link with PHC^(1,5)^.

The use of a taxonomy framework with standardized language to explain the problem, the expected outcomes and nursing interventions is a strategy to reduce communication noise, favoring the sharing of information and professional knowledge and inter-institutional benchmarking nationally and internationally, which justifies the choice of NNN taxonomy^(10-12)^. The protocol presented met the NP steps provided for in Resolution 358/2009^(8)^, namely: nursing data collection (or NH); nursing diagnosis; nursing planning; nursing implementation and assessment. The alignment between the steps was favored by the connection between NNN taxonomies^(10-12)^.

The documentation of nursing actions is provided for in Resolution 429/2012^(24)^, whether in traditional or electronic format. Nursing records favor communication between members of the nursing team, the multidisciplinary team and the RAS, for continuity of care. Moreover, through the records, action duplicity is avoided, enabling managing care costs, composing the user’s health record, allowing the monitoring of their health-disease situation, as well as preventing therapeutic interactions resulting from multiple treatments peculiar to the aging process^(29-30)^.

In this sense, the protocol presented supports the systematization of care for the investigated population, focusing on disease prevention, disease reduction and early diagnosis in the PHC context. The use of protocols favors nurses’ work, guiding therapeutic conducts. This recommendation is justified by its ability to synthesize the therapeutic process, combined with time spent reduction to record their professional conduct, adding standardization of information available for continuity of nursing care.

### Study limitations

The study limitation lies in the fact that it was carried out during the COVID-19 pandemic, making steps 10 to 12 of the A Step-by-Step Guide to Developing Protocols unfeasible. It is suggested to validate steps 10 to 12 in printed and electronic formats.

### Contributions to health

The PECPIV-DRC protocol is a contribution to PE in PHC in CKD prevention and the aggravation of associated comorbidities.

## CONCLUSIONS

The protocol developed is a proposal that makes it possible to operationalize the NP based on Neuman’s stressors, which guides the care and nursing records planned and performed on scientific and technical bases, using taxonomic, conceptual and care frameworks in nursing care for older adults vulnerable to CKD in PHC.
